# Complex modulation of androgen responsive gene expression by methoxyacetic acid

**DOI:** 10.1186/1477-7827-9-42

**Published:** 2011-03-31

**Authors:** Gargi Bagchi, Yijing Zhang, Kerri A Stanley, David J Waxman

**Affiliations:** 1 Division of Cell and Molecular Biology, Department of Biology, Boston University, Boston, MA 02215, USA

## Abstract

**Background:**

Optimal androgen signaling is critical for testicular development and spermatogenesis. Methoxyacetic acid (MAA), the primary active metabolite of the industrial chemical ethylene glycol monomethyl ether, disrupts spermatogenesis and causes testicular atrophy. Transcriptional *trans*-activation studies have indicated that MAA can enhance androgen receptor activity, however, whether MAA actually impacts the expression of androgen-responsive genes *in vivo*, and which genes might be affected is not known.

**Methods:**

A mouse TM3 Leydig cell line that stably expresses androgen receptor (TM3-AR) was prepared and analyzed by transcriptional profiling to identify target gene interactions between MAA and testosterone on a global scale.

**Results:**

MAA is shown to have widespread effects on androgen-responsive genes, affecting processes ranging from apoptosis to ion transport, cell adhesion, phosphorylation and transcription, with MAA able to enhance, as well as antagonize, androgenic responses. Moreover, testosterone is shown to exert both positive and negative effects on MAA gene responses. Motif analysis indicated that binding sites for FOX, HOX, LEF/TCF, STAT5 and MEF2 family transcription factors are among the most highly enriched in genes regulated by testosterone and MAA. Notably, 65 FOXO targets were repressed by testosterone or showed repression enhanced by MAA with testosterone; these include 16 genes associated with developmental processes, six of which are *Hox *genes.

**Conclusions:**

These findings highlight the complex interactions between testosterone and MAA, and provide insight into the effects of MAA exposure on androgen-dependent processes in a Leydig cell model.

## Background

Androgen signaling is critical for development of the male sexual phenotype, maturation of secondary sex characteristics and maintenance of muscle mass and bone density [1]. Disruption of androgen signaling can lead to a spectrum of developmental problems in male sexual characteristics and reproductive behavior [2]. Androgen action is mediated by androgen binding to androgen receptor (AR), a ligand-activated transcription factor that binds genomic regulatory elements associated with androgen responsive genes [3]. AR binding sites are often far (>10 kb) from transcription start sites of androgen-regulated genes, and many AR binding sites contain non-canonical androgen response elements [4-6]. Many transcription factors interact with AR, including GATA factors [7], STAT5 [8], NF1 and SP1 [9], which can increase AR transcriptional activity, as well as Forkhead proteins [10-12], P53 [13] and LEF/TCF factors [14], which are reported to exert both repression and enhancement of AR transcriptional activity. Some of these effects may involve local interactions, as binding sites for GATA and Forkhead, as well as OCT family factors are often enriched nearby AR binding sites [4-6]. These findings suggest that physiological or pathophysiological conditions that affect the expression or activity of AR-interacting transcription factors such as these may impact AR activity.

Many foreign chemicals can modulate AR activity; these include drugs and environmental chemicals that bind directly to AR and antagonize its transcriptional activity [15,16]. AR activity can also be modulated by foreign chemicals that exert effects on AR indirectly, *via *intracellular signaling [17,18]. One example is methoxyacetic acid (MAA), a testicular toxicant and the primary, active metabolite of the industrial chemical ethylene glycol monomethyl ether [19,20]. MAA enhances the transcriptional activity of several nuclear receptors [21,22], including AR [19,22-24], by a mechanism that involves tyrosine kinase activity and requires PI3-kinase signaling [22]. The inappropriate enhancement of AR transcriptional activity by MAA could contribute to the testicular toxicity associated with MAA exposure, given the importance of AR in somatic cells of the testis for spermatocyte survival [25].

Earlier studies of the potentiation of AR transcriptional activity by MAA used AR reporter gene assays to demonstrate enhancement of androgen response [22]. However, while reporter gene assays are an important tool for studying gene regulation, transfected reporter gene constructs do not always reflect the regulation of endogenous genes in untransfected cells *in vivo*. Moreover, in the case of MAA, artefactual effects on the CMV promoter used in one study to express estrogen receptor required for reporter gene activity were reported [26]. It is therefore important to determine the effects of MAA on the expression of endogenous androgen responsive genes to determine whether MAA can, indeed, potentiate androgen responses, to identify the specific genes whose expression is affected, and to elucidate the nature and extent of interactions between MAA and androgen, both positive and negative.

In this study, we develop an androgen-responsive mouse testicular Leydig cell line, TM3-AR, and use it to investigate the impact of MAA on androgen responsive gene expression by global transcriptional profiling. Our findings reveal that MAA alters the expression of large numbers of testosterone-responsive genes. We also find that the androgenic environment can influence the effects of MAA on gene expression, with many examples of both stimulatory and inhibitory interactions between MAA and testosterone. Motif analysis identified binding sites for transcription factors whose putative targets are enriched in genes showing either positive or negative interactions between MAA and testosterone, providing further insight into the mechanisms that govern these gene interactions. Enriched micro-RNA binding sites in the 3'-untranslated region (3'-UTR) of target genes were also identified. These findings demonstrate that the impact of MAA on androgen gene responses is complex and suggest target genes and pathways through which MAA may exert toxicity to somatic cells of the testis.

## Methods

### Chemicals and reagents

MAA, horse serum and testosterone were purchased from Sigma Chemical Co, St. Louis, MO. DMEM-F12 culture medium, fetal bovine serum (FBS), HEPES buffer and TRIzol reagent were purchased from Invitrogen Corp. (Carlsbad, CA).

### Cell culture and TM3-AR cell preparation

Mouse TM3 Leydig cells and LNCaP cells were obtained from American Type Culture Collection, Manassas, VA. TM3 and TM3-AR cells (see below) were grown in DMEM-F12 medium containing 5% horse serum and 2.5% FBS. LNCaP cells were maintained in RPMI 1640 containing 10% FBS. RNA was isolated using TRIzol reagent using the manufacturer's protocol. Mouse TM3 cells stably expressing human AR cDNA were prepared by retroviral infection of TM3 cells, as follows. The coding sequence of AR was excised from plasmid pSV-ARO (Dr. A.O. Brinkmann, University Medical Center Rotterdam, The Netherlands) and subcloned by blunt end ligation into the retroviral plasmid vector pWZL-Blast (Dr. D. White, Millenium Pharmaceuticals, Cambridge MA) to yield pWZL-Blast-AR. pWZL-Blast is based on the pBabe plasmid [27] and encodes a blasticidin-resistance gene transcribed from the retroviral long terminal repeat. Retroviral particles were generated as described [28] by transfecting the packaging cell line HEK293 with pWZL-Blast-AR. Culture medium containing retroviral particles was collected 48 h later and applied to TM3 cells. Pools of blasticidin-resistant cells were selected for 4 days using blasticidin *S*-hydrochloride and then verified as expressing AR by qPCR.

To obtain samples for microarray analysis, TM3-AR cells were treated for 24 hr with either testosterone (10 nM) or MAA (5 mM), or with testosterone in combination with MAA. The concentration of testosterone was chosen to saturate AR, and the concentration of MAA was chosen based on considerations described in our earlier studies [22,29], and based on its correspondence to the plasma concentration associated with ethylene glycol monomethyl ether-induced germ cell toxicity in mice [30]. The concentration of MAA used did not alter the cell growth rate or cause any loss of cell viability over the course of at least 48 hr. RNA was isolated and validated by RNA integrity number >8.5, as determined using an Agilent Bioanalyzer 2100 instrument (Agilent Technologies, Santa Clara, CA).

### qPCR

Total RNA isolated from treated or untreated cultured cells was incubated with RQ1 RNAse-free DNAse for 1 h at 37°C followed by heating at 75°C for 5 min. cDNA synthesis and qPCR analysis using SYBR Green I-based chemistry were performed as described [31]. Dissociation curves were examined after each qPCR run to ensure amplification of a single, specific product. qPCR primers were designed using Primer Express software (Applied Biosystems) and are shown in Additional file 1, Table S1. Relative RNA levels were calculated after normalization to the 18S rRNA content of each sample using the comparative Ct method, under conditions where the Ct number is in its log2 linear range.

### Microarray analysis

Each RNA sample used for microarray analysis was a pool prepared from three independent TM3-AR cell cultures (three different passages), each treated as described above. Two such pools of TM3-AR cell RNA (representing a total of 5 independent treated cell cultures) were prepared and used in two independent sets of microarrays, each of which represented 3 of the 5 independent cultures. Each set of microarrays was comprised of four separate competitive hybridization arrays (i.e., four microarray experiments): testosterone vs. control, MAA vs. control, testosterone + MAA vs. testosterone, and testosterone + MAA vs. MAA. This approach, employing pools of biological replicates, minimizes the impact of culture-to-culture variations that are unrelated to the treatments *per se*. cDNAs transcribed from each pooled RNA sample were labeled with Alexa 647 or Alexa 555 dyes in a fluorescent reverse pair design (dye swap) for competitive hybridization to Agilent Whole Genome Mouse Microarrays (Agilent Technology, array platform G4122F). Sample labeling, hybridization to microarrays, scanning, analysis of TIFF images using Agilent's feature extraction software, calculation of linear and LOWESS normalized expression ratios and *p*-value calculation using Rosetta Resolver (version 5.1, Rosetta Biosoftware) were carried out as described [32,33]. For dye swapping experiments, the Alexa 555-labeled RNA from one of the treatment conditions (testosterone and/or MAA treated) was mixed with Alexa 647-labeled RNA for the appropriate reference control (as specified above), and vice versa. Features flagged as saturated in both fluorescence channels or flagged as non-uniformity outliers in either channel were excluded from analysis. The full set of normalized expression ratios and *p*-values is available at the Gene Expression Omnibus web site of NCBI [34] as GEO series GSE27410.

### Microarray annotation and statistical analysis

Agilent mouse microarray G4122F contains 41,174 mouse probes (features), each 60-nt in length. Accession numbers were obtained for 39,355 out of the 41,174 probes, of which 33,011 were assigned gene names. An additional 3,570 probes were assigned gene names using the microarray probe annotation tool AILUN [35], which maps microarray probes to Entrez genes. Each probe corresponding to a distinct mouse transcript is referred to as representing a separate gene/gene product. For each microarray probe, a mean fold-change and *p*-value was calculated based on the set of microarray expression ratios using the Rosetta Resolver-based error model [32]. The error model uses technology-specific data parameters to stabilize intensity variation estimates, along with error-weighted averaging of replicates. This approach has been demonstrated to provide an effective increase in statistical power [32]. The statistical significance of differential expression of each gene was determined by application of a filter (*p *< 0.005) to the Rosetta-generated *p-*values. Next, a |fold-change| filter of >2-fold was combined with the above *p*-value filter to determine the number of probes that were differentially regulated in any of the four microarray experiments. In total, 6,416 probes met the combined thresholds for differential expression (|fold-change| >2) and statistical significance (*p *< 0.005) in at least one of the four experiments. In those cases where two or more differentially expressed probes mapped to the same gene and gave the same pattern of expression across all four microarrays (reflecting probe redundancy in the array platform), a single representative probe was retained in the final data set. A total of 884 redundant probes were thus eliminated, giving a total of 5,532 non-redundant probes that met the threshold criteria for both differential expression (|fold-change| >2) and statistical significance (*p *< 0.005) in at least one of the four experiments (Additional file 2, Table S2A). The number of probes expected to meet the combined threshold by chance is 0.005 × 6,416, or 32 probes. The actual number of probes meeting the combined threshold was 7,811, corresponding to an apparent false discovery rate of 32/7,811, or 0.41%. Commonly used multiple testing correction methods such as Bonferroni or Holm step-down were not applied as these eliminate a large number of true positives and introduce an inappropriate overcorrection.

A system of binary and decimal flags, termed total flag sum (TFS), was used to cluster the differentially regulated genes into subgroups based on their patterns of expression across the four microarray experiments [36]. Briefly, all genes that met the above fold-change and *p*-value threshold criteria for one or more of the four microarray experiments were assigned a binary flag value of 1, 2, 4 and 8 respectively. The sum of these binary flag values defines the whole number portion of the flag assigned to each gene and indicates which of the four microarrays met the specified threshold criteria in our analysis. In addition, decimal values of 0.1, 0.01, 0.001 and 0.0001, or 0.2, 0.02, 0.002, and 0.0002 were respectively assigned to each of the four microarrays to indicate the direction of regulation of the genes in the array (decimal flags with values of 1 indicate up-regulation, whereas those with a value of 2 indicate down-regulation). Thus, for each gene, the TFS group designation, comprising the binary sum plus the decimal values, indicates which of the four arrays met the threshold criteria for inclusion and the direction of regulation, as outlined in Additional file 2, Table S2B. As an example, the 472 genes in TFS group 9.1001 (see Table 1, below) all meet the combined threshold for up regulated expression in array experiments 1 and 4 (testosterone vs. control, and testosterone + MAA vs. MAA, respectively), but not in array experiments 2 and 3 (MAA vs. control, and testosterone + MAA vs. testosterone). The whole number portion of the TFS group number, 9, equals the sum of the binary flag values 1 + 8, i.e., significant regulation on the 1^st ^and 4^th ^array experiments. Similarly, TFS group 6.0220 indicates down regulation in the 2^nd ^and 3^rd ^array experiments, etc.

**Table 1:** Classification of TFS groups of genes responding to testosterone and/or MAA

Class	Description	TFS group	TFS Gene Count	Response to			
				T	MAA	T/MAA vs. T	T/MAA vs. MAA
I	No interaction between T and MAA	9.1001	472	Up	-	-	Up
		9.2002	492	Down	-	-	Down
		6.0110	350	-	Up	Up	-
		6.0220	174	-	Down	Down	-
		3.1100	53	Up	Up	-	-
		3.2200	55	Down	Down	-	-
		15.2112	59	Down	Up	Up	Down
IIa	MAA enhances action of T	8.0001	421	-	-	-	Up
		8.0002	398	-	-	-	Down
		14.0111	60	-	Up	Up	Up
		14.0222	24	-	Down	Down	Down
IIb	T enhances action of MAA	4.0010	355	-	-	Up	-
		4.0020	264	-	-	Down	-
		13.1011	144	Up	-	Up	Up
		13.2022	56	Down	-	Down	Down
IIc	T enhances action of MAA, and MAA enhances action of T	15.1111	77	Up	Up	Up	Up
		15.2222	39	Down	Down	Down	Down
		12.0011	316	-	-	Up	Up
		12.0022	81	-	-	Down	Down
IIIa	MAA blocks action of T	1.1000	329	Up	-	-	-
		1.2000	307	Down	-	-	-
IIIb	T blocks action of MAA	2.0100	302	-	Up	-	-
		2.0200	165	-	Down	-	-
IIIc	MAA blocks the action of T, but MAA alone shows no activity	5.1020	62	Up	-	Down	-
		5.2010	52	Down	-	Up	-
IIId	T blocks the action of MAA, but T alone shows no activity	10.0102	107	-	Up	-	Down
		10.0201	16	-	Down	-	Up

Regulated genes were clustered based on whether there is an interaction between testosterone and MAA, as follows: Class I represents genes that respond to testosterone or to MAA with no interactive effect. Class II represents genes whose response to testosterone was enhanced by MAA (IIa), genes whose response to MAA was enhanced by testosterone (IIb), and genes whose responses to testosterone and MAA showed mutual enhancement (IIc). Class III corresponds to genes whose response to testosterone could be blocked by MAA (IIIa, IIIc) or whose response to MAA could be blocked by testosterone (IIIb, IIId). See Additional file 2, Table S2C for further details.

### Gene Ontology (GO) and motif enrichment analysis

GO term enrichment analysis for each TFS group was carried out using DAVID data sets [37]. Briefly, genes in each TFS group were iteratively compared with genes in each gene set that share a common GO term, and the number of overlapping genes was used to calculate an enrichment score and a Fisher's exact test *p-*value for each TFS group and each gene set. GO terms enriched at *p *< 0.001 and containing >5 genes with the specific GO term for at least one TFS group were selected, and TFS groups with at least one enriched GO term were selected. A total of 156 unique GO terms enriched in 17 TFS groups were obtained (Additional file 3, Table S3A). Hierarchical clustering was implemented using Cluster [38], and a corresponding heat map was drawn using Java Treeview [39]. Cis-regulatory elements associated with the gene expression changes induced by testosterone and MAA were identified by gene set enrichment analysis (GSEA) by searching each group of genes against the 836 motif gene sets and against the 221 predicted microRNA (miRNA) target gene sets that comprise the C3 module of the Molecular Signatures database [40]. The motif gene sets contain genes sharing a cis-regulatory motif conserved across the human, mouse, rat, and dog genomes, and the motifs represent known or likely transcription factor binding sites in a 4 kb genomic region centered on the transcription start site of each gene. The miRNA target gene sets are comprised of genes with the corresponding miRNA binding sites present in 3'-UTR sequences.

## Results

### Generation of TM3-AR cells and AR expression

**Figure 1:**
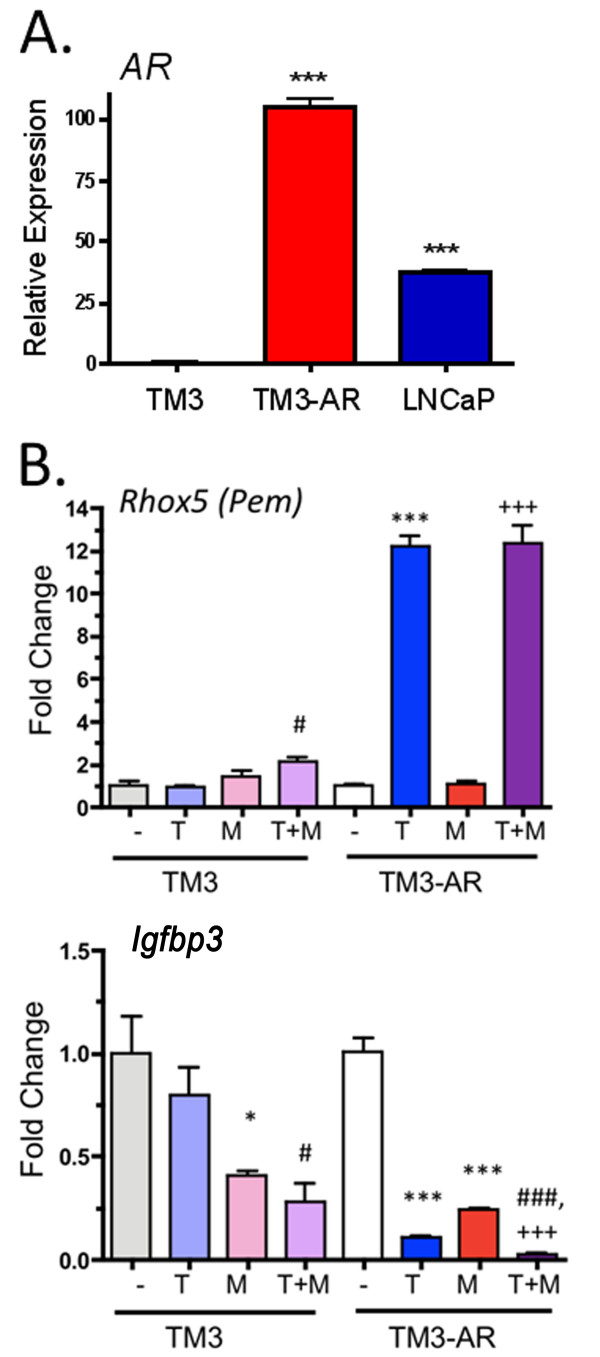
**qPCR analysis of the expression of *AR *(A), testosterone induced gene *Pem *(B) and testosterone repressed gene *Igfbp3 *(B)**. In A, infection of TM3 cells with retorvirus expressing AR cDNA is shown to lead to AR expression at a level similar to that of LNCaP prostate cancer cells. Significantly different from TM3 cells at *p *< 0.001 (***). In B, cells were untreated (DMSO vehicle alone, -), or were treated for 24 hr with testosterone (T, 10 nM), MAA (M, 5 mM), or with T + MAA, as indicated. *Rhox5 *is a testosterone-inducible Sertoli cell marker gene that is also expressed at a low level in Leydig cells *in vivo *[56]. *Igfbp3 *is repressed by testosterone, and by MAA [22], with the latter response also seen in the TM3 cells deficient in AR. Data are mean ± SD values based on n = 3 replicates, with the untreated TM3 cell value set to 1.0. Significantly different from untreated control at *p *< 0.05 (*) or at *p *< 0.001 (***); significantly different from T at *p *< 0.05 (#) or at *p *< 0.001 (###); and significantly different from MAA at *p *< 0.001 (+++).

TM3 mouse Leydig cells are reported to be MAA responsive [19], however, we found AR expression to be very low, and correspondingly, the androgen responsiveness of these cells was very weak, as judged by qPCR analysis (Figure 1). To increase the androgen response, AR cDNA was stably transfected into TM3 cells using a retroviral vector. The resulting pool of TM3-AR cells showed a marked increase in AR expression, comparable to that of the widely studied androgen responsive cell line LNCaP (Figure 1A). The androgen responsiveness of TM3-AR cells was confirmed by the ~5-fold increase in expression of *Rhox5 *(*Pem) *and by the ~10 fold decrease in expression of *Igfbp3 *following testosterone treatment; neither gene showed a significant response to testosterone in TM3 cells, but the repression of *Igfbp3 *by MAA [22] was evident in both cell lines (Figure 1B).

### Impact of MAA on TM3-AR cell gene expression

**Figure 2:**
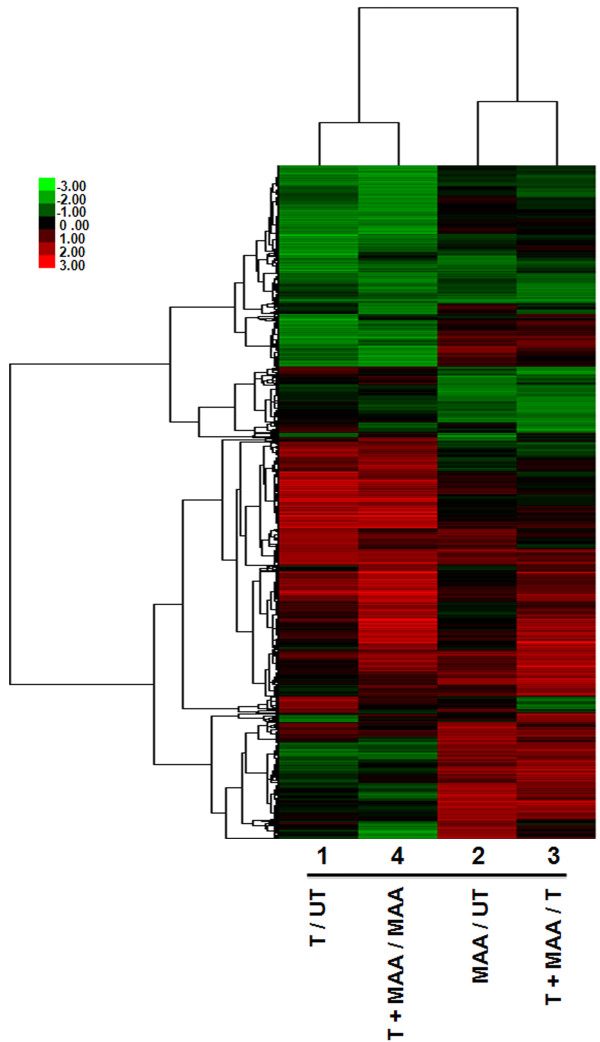
**Heat map displaying the effects of testosterone, MAA or both in combination on TM3-AR cell gene expression**. Shown is the hierarchical clustering heat map of 5,532 responsive genes based on log2 ratios, with the scale as shown at the bottom, right. T, testosterone; UT, untreated.

**Figure 3:**
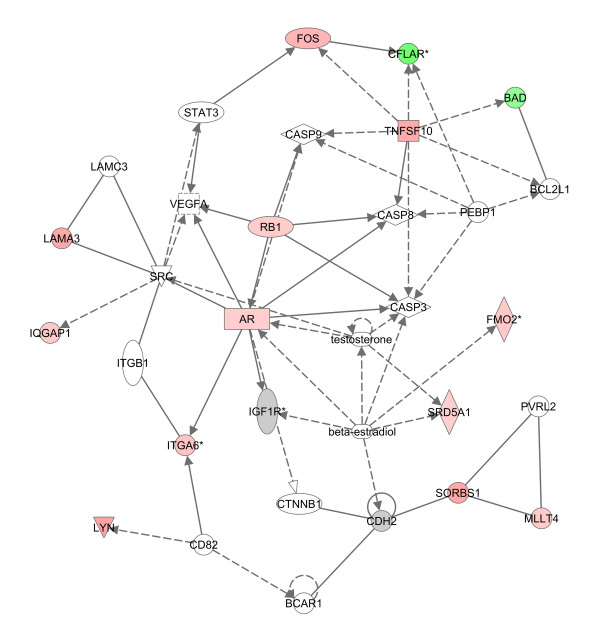
**Top network affected by MAA**. This network was identified by Ingenuity Pathway Analysis, and involves cell death, cellular development and cell-to-cell signaling and interaction. Dashed arrows indicate regulation of gene expression, arrows with solid lines represent protein-DNA interactions, and solid lines indicate protein-protein interactions. Lines in blue identify factors and processes directly connected to testosterone.

The global impact of MAA on androgen-responsive gene expression was evaluated by microarray analysis. TM3-AR cells were treated for 24 h either with testosterone, MAA, a combination of testosterone and MAA, or vehicle control. Total RNA from each group was then analyzed on whole-mouse genome two-color expression microarrays for the following four comparisons: Array 1, testosterone vs. control; Array 2, MAA vs. control; Array 3, testosterone + MAA vs. testosterone; Array 4, testosterone + MAA vs. MAA. Normalized expression ratios and *p*-values were determined, and genes meeting our combined threshold for significance (|fold-change| > 2 and *p *< 0.005; see Methods) for at least one of the four microarray comparisons were identified. A total of 5,532 genes of interest were thus obtained after elimination of redundant probes. Hierarchical clustering of these 5,532 genes revealed closest correlation between arrays 1 and 4 (effects of testosterone in the absence and presence of MAA, respectively), and, to a lesser extent, between arrays 2 and 3 (effects of MAA in the absence and presence of testosterone, respectively) (Figure 2). A complete listing of these genes, along with their expression ratios, measured signal intensities and gene annotations is provided in Additional file 2, Table S2A. Testosterone induced 1,233 genes and repressed 1,205 genes (array 1), while MAA induced 1,206 genes and repressed 525 genes (array 2). The combination of testosterone + MAA induced 1,553 genes and repressed 748 genes when compared with testosterone treatment alone (array 3), while 1,587 genes were induced and 1,396 genes were repressed by testosterone + MAA, when compared with MAA treatment alone (array 4). Among the genes induced by testosterone were *Rhox5 *(*Pem*) [41] and *Amotl1 *[42], two well-characterized androgen-inducible genes. 87% of the MAA-responsive genes in TM3-AR cells identified on array 2 overlap with the set of MAA-responsive genes that we previously identified in TM3 cells that do not express AR [29], validating the robustness of the MAA response. Ingenuity Pathway Analysis revealed testosterone related gene networks that respond to MAA; these include cell death and cellular development, reproductive system disease, and small molecule biochemistry (Figure 3 and Additional file 4, Figure S1).

### MAA affects androgen response in multiple ways: clustering by significance and differential expression

The impact of MAA on testosterone gene responses was investigated by classification of the regulated genes using a binary flagging system [36], whereby each gene was assigned to a specific category, termed TFS (total flagging sum), based upon its expression ratio and *p-*value in each of the four microarray experiments (Additional file 2, Table S2B). This system provides a simple way to identify gene groups that responded to testosterone or MAA and to determine whether there is any interaction between them. Of the 5,532 genes of interest, 5,230 (95%) could be grouped into four major classes based on the interactions of testosterone and MAA (Table 1 and Additional file 2, Table S2C). Class I is comprised of 1,655 genes (30% of the total) distributed into 7 TFS gene groups. These genes responded to testosterone and/or MAA but showed no interaction between testosterone and MAA. Class II is comprised of 2,235 genes (40%) distributed into 12 TFS groups. These genes displayed positive interactions between testosterone and MAA, i.e., testosterone enhanced responses to MAA, and/or vice versa, or the combination of both agents induced gene responses not observed with the individual treatments. Class III is comprised of 1,240 genes (24%) distributed into 8 TFS groups. These genes showed negative interactions between testosterone and MAA, i.e., the response to testosterone could either be blocked or reversed by MAA, or vice versa. The remaining 302 genes were distributed into 25 small TFS groups and were not considered further (Additional file 2, Table S2C).

It should be noted that the induction or repression observed by treating with testosterone + MAA is being compared with that obtained with testosterone alone (array 3) or to MAA alone (array 4), and not to the vehicle-treated control. In case of class III genes, this is of particular importance, as in some cases, testosterone alone may cause gene induction, while treatment with testosterone + MAA might cause repression relative to the level of expression with testosterone alone but not when compared to vehicle control. For example, in case of *Cep70 *in TFS group 5.2010, the microarray signal intensities (corresponding to expression levels) in the control, testosterone, and testosterone + MAA samples were 6,815, 3,303 and 7,092, respectively (Additional file 2, Table S2A). These values indicate repression by testosterone and induction by testosterone + MAA as compared to testosterone, but not when compared to vehicle control. The net result, however, is that MAA blocks the repressive action of testosterone. Patterns such as these, where testosterone or MAA block or reverse the response to the other agent, characterize the genes in class III.

### Real time qPCR validation

**Figure 4:**
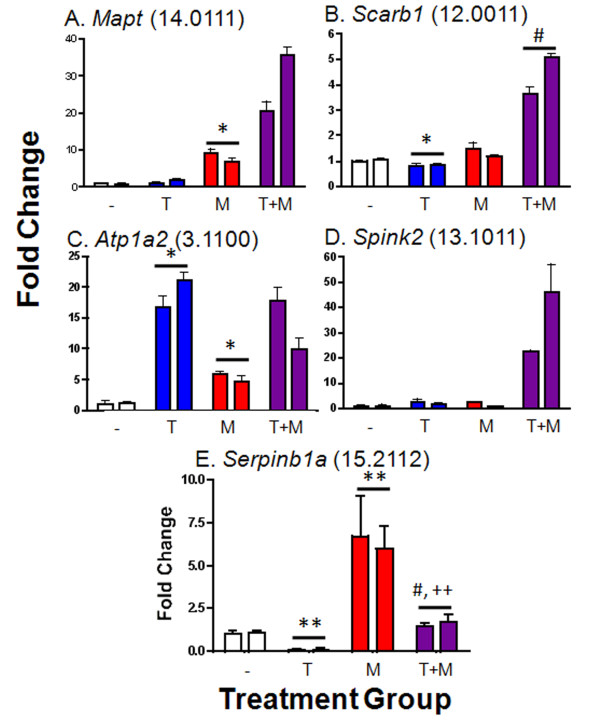
**qPCR validation of microarray results for genes representative of five TFS groups**. TM3-AR cells were treated with vehicle (DMSO, -), testosterone (T), MAA (M), or with T + MAA (T+M), as in Figure 1B. qPCR analysis was carried out for the five indicated genes; their TFS group assignments (Additional file 2, Table S2) are shown in parentheses. Two pools of TM3-AR cell RNA, each comprised on RNA isolated from 3 independent cell cultures, were assayed and are represented by the pair of bars in each treatment group. Data are mean ± SD values based on n = 3 replicates, with the first vehicle control pooled sample set to 1.0. Significantly different from control at *p *< 0.05 (*) or at *p *< 0.01 (**); significantly different from T at *p *< 0.05 (#); and significantly different from MAA at *p *< 0.01 (++). qPCR primers used for this analysis are shown in Additional file 1, Table S1.

To confirm the results of the microarrays, qPCR analysis was carried out for 15 genes representing five different TFS groups (Figure 4 and Additional file 5, Table S4). Results were in close agreement, although in several cases fold-change values determined by qPCR were greater than those obtained by microarray (e.g., 38.6-fold induction of *Tulp2 *by testosterone + MAA vs. testosterone alone by qPCR, vs. 7.6-fold induction by microarray; Additional file 5, Table S4). This finding is consistent with the compression of expression ratios commonly seen using microarrays.

### Functional impact of MAA on androgen responsive gene expression

**Figure 5:**
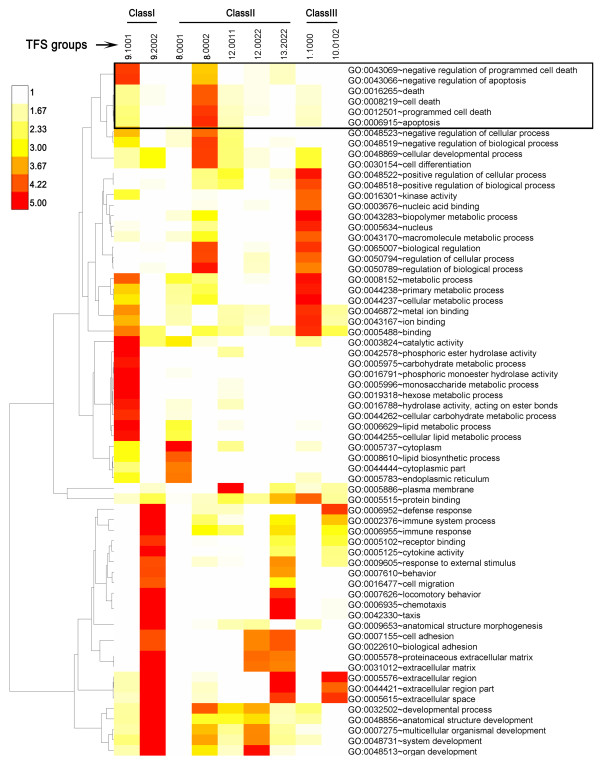
**GO term enriched in TFS groups**. Shown is a hierarchical clustering of GO terms that were significantly enriched (*p *< 0.0001, and number of included genes >10) in at least one TFS group. GO terms are displayed at the right and TFS group numbers are shown across the top. The color bar at the top left represents -log10 *p-*values, with higher numbers (darker colors) indicating more significant enrichment. A complete list of enriched GO terms, *p-*values, enrichment scores and regulated genes in each GO term is provided in Additional file 2, Table S2A. GO terms associated with apoptosis are in the boxed region at the top. TFS groups represent the following groups of genes 9.1001, genes induced by testosterone, both without and with MAA present; 9.2002, genes suppressed by testosterone, both without and with MAA present; 8.0001, genes induced by testosterone, but only in the presence of MAA; 8.0002, genes suppressed by testosterone but only in the presence of MAA; 12.0011, genes induced by testosterone + MAA, relative to testosterone alone and relative to MAA alone; 12.0022, genes suppressed by testosterone + MAA, relative to testosterone and relative to MAA alone; 13.2022, genes suppressed by testosterone either alone or in combination with MAA, and MAA enhances the suppression by testosterone; 1.1000, genes whose induction by testosterone is blocked by MAA; and 10.0102, genes induced by MAA, but only in the absence of testosterone, and suppressed by testosterone, but only in the presence of MAA.

Gene Ontology (GO) term analysis was carried out to identify the functional gene categories (i.e., the GO terms) enriched in the sets of genes that comprised each major TFS group. These analyses were useful for elucidating the functional consequences of testosterone and MAA treatment and their interactions. A summary of the major results is presented in Figure 5, with full details provided in Additional file 3, Table S3A. Among the gene groups showing no interaction between MAA and testosterone (class I genes), 964 genes responded to testosterone but not to MAA. The 472 class I genes up regulated by testosterone (TFS group 9.1001) were most highly enriched in GO terms associated with negative regulation of apoptosis, ion binding and lipid metabolism (Figure 5). In contrast, the 492 class I genes down regulated by testosterone (TFS 9.2002) were enriched for immune response, cytokine activity, chemotaxis and extracellular matrix and developmental processes (Figure 5). Class II genes, whose responses are enhanced by testosterone and/or MAA, were distributed into three subclasses (Table 1), based on whether MAA enhanced responses to testosterone (class IIa, 819 genes), testosterone enhanced responses to MAA (class IIb, 619 genes), or the enhancement was mutual (class IIc, 734 genes). Class IIa genes showed the highest enrichment for lipid biosynthesis (TFS 8.0001), apoptosis, cell differentiation, and regulation of biological processes (TFS 8.0002). Class IIb genes showed highest enrichment for extracellular matrix, cell adhesion and chemotaxis (TFS 13.022), while class IIc genes showed highest enrichment for plasma membrane (TFS 12.0011) and for extracellular matrix, cell adhesion, and organ development (TFS 12.0022). Class III genes were distributed into subclasses, based on whether MAA blocked the response to testosterone (IIIa, IIIc) or testosterone blocked the response to MAA (IIIb, IIId) and whether testosterone and MAA are active alone (IIIa, IIIb), or not (IIIc, IIId). The largest TFS group in class IIIa (TFS 1.1000; 329 genes) showed greatest enrichment for cellular and biopolymer metabolic processes, nucleic acid binding, kinase activity and metal ion binding, and included 55 genes that encode nuclear factors, indicating a wide range of impact of MAA on testosterone responses. Finally, the genes in class IIId, TFS group 10.0102, whose induction by MAA was blocked by testosterone, and whose suppression by testosterone was only manifested when MAA was present, showed greatest enrichment for extracellular region and defense response (Figure 5 and Additional file 3, Table S3A).

### Motif enrichment analysis

Species-conserved transcription factor binding site motifs and 3'-UTR miRNA binding sites enriched in the genes belonging to each TFS group were identified by GSEA [40], as described under Methods. A total of 64 motifs enriched in 13 TFS groups were identified after filtering out motifs not showing enrichment at *p *< 0.001 and TFS groups with no enriched motifs at *p *< 0.001. Table 2 summarizes the results, and the discovered motifs and miRNA binding sites are clustered in Additional file 6, Figure S2. Detailed information for each motif and miRNA-binding site, including enrichment scores, is provided in Additional file 3, Table S3B. Motifs enriched in the promoters of class I genes in TFS group 9.2002 including binding sites for FOXO and other forkhead family transcription factors, with FOXO motifs showing the most significant enrichment (*p *= 3.2 E06). Six *Hox *genes that were repressed by testosterone (TFS 9.2002) or whose repression was mutually enhanced by MAA and testosterone (TFS 12.0022), i.e., *Hoxb5, Hoxb9, Hoxc6, Hoxc*8, *Hoxd3 *and *Hoxd13*, are putative targets of FOXO (Additional file 2, Table S2D), suggesting an important role for *Fox *and *Hox *genes in the modulation of AR activity by MAA. Motifs for MEF2A, TCF/LEF and STAT5 factors were also enriched among the class I TFS groups, as were 3'-UTR binding sites for several miRNAs. The enriched motifs for class II genes, whose response to testosterone and MAA showed mutual enhancement, include binding sites for MEF2, HOXA4, E2F, TCF, GATA, and others, distributed among several TFS groups (Table 2). Binding sites for STAT5, TCF/LEF, FOXF2, PGR, MYCN and several miRNAs were enriched in class III (TFS groups 1.1000, 2.0100 and 2.0200). These analyses also revealed a large number of miRNA binding sites that are common to the 3'-UTR sequences of testosterone-regulated genes.

**Table 2:** Transcription factors and miRNAs showing enrichment and potentially contributing to expression responses in the indicated gene classes and TFS groups.

Class	TFS group	Motif name	miRNA name
I	9.1001	MEF2A	-
	9.2002	FOXO, LEF1, STAT5B, POU1F1, NFAT, TLX-2, GATA-1, STAT5a, MAZ, IRF-7, TAF, FOXF2, JUN, FOXA1, FOXJ1, ZHX2	MIR-23B, MIR-144, MIR-142
	6.0110	MEF2A	-
	15.2112	TGIF	-
IIa	8.0002	E2F, TCF3, ETS-2, PAX4	-
IIb	4.0010	MEF2A	-
	4.0020	HOXA4, GCM1, RFX1, GATA3	MIR-24
	13.2022	TAF	-
IIc	12.0022	TCF8, FOXO, TAF	MIR-524
IIIa	1.1000	STAT5B, STAT5A, PGR, LEF1, TCF3, FOXF2, E4F1	MIR-124A, MIR-17-5P, MIR-20A, MIR-106A, MIR-106B, MIR-20B, MIR-519D", MIR-182, MIR-200B, MIR-200C, MIR-429, MIR-202, MIR-199A, MIR-519E, MIR-9
IIIb	2.0100	MYCN, OLF1, MYOD1	-
	2.0200	-	MIR-493
-	11.1101	LEF1	-

Motifs and miRNAs within each TFS group are listed in order of decreasing enrichment *p*-value, based on data provided in Additional file 3, Table S3B.

## Discussion

MAA, the active metabolite of the industrial chemical ethylene glycol monomethyl ester, is an established testicular toxicant. Earlier studies suggested that MAA could potentiate AR transcriptional activity without significantly altering the dose-response curve for androgen activity, as determined in reporter gene studies [22]. Presently, the impact of MAA on expression of androgen-regulated genes was characterized globally in a mouse Leydig cell model. Mouse TM3 cells stably expressing AR were treated with testosterone, MAA, or with both chemicals in combination, and 5,532 genes responding to one or more treatments were identified and then classified, and sub-classified, based on their patterns of response to each treatment. GO term and motif enrichment analysis were carried out for genes in each subgroup to help identify the biological functions and pathways affected by MAA as it impacts cellular responses to testosterone.

### MAA has a wide range of impact on androgen responses

AR activated by testosterone can have direct effects on target gene transcription [43], as well as indirect effects mediated by intracellular signaling pathways. These range from stimulation of protein kinases, to direct modulation of voltage- and ligand-gated ion channels and transporters [44], some of which may lead to changes in gene expression [44]. Here, our microarray analysis identified large numbers of genes that responded to testosterone, a subset of whose responses were modulated by MAA. These genes contribute to a wide range of biological processes, including cell death, development, ion binding, kinase activities and transcription. These findings may help explain some of the previous findings about the toxicities of MAA. For example, MAA stimulates apoptosis of male germ cells [19,23,45-48] by mechanisms proposed to involve various kinases and ion transporters [45,47]. Ion transport is important for the maintenance of intracellular pH, perturbation of which can affect germ cell fertility [49]. Proteins involved in transport comprise a large group of MAA regulated genes, including genes whose expression is affected by MAA alone, and genes that are additively or synergistically regulated by MAA and testosterone. For instance, GO term enrichment analysis identified 52 ion binding protein and 19 kinase genes that were significantly enriched in the set of genes induced by testosterone whose induction is blocked by MAA (TFS group 1.1000). This same gene set showed enrichment for genes that negatively regulate apoptosis. In contrast, both positive and negative regulators of apoptosis were enriched in the set of genes repressed by testosterone in the presence of MAA (TFS group 8.0002). Further investigation will be required to determine whether these gene responses contribute to the testicular toxicities of MAA seen in mouse models, as well as their relevance to humans exposed to MAA.

### Transcription factors involved in MAA modulation of testosterone response

TM3-AR cells showed complex patterns of response to testosterone and MAA (Table 1), indicating that multiple mechanisms likely contribute to MAA modulation of responses to testosterone, and to testosterone modulation of responses to MAA. One mechanisms could involve effects of MAA on the expression of AR, whose levels were increased ~2-fold by MAA, both in the absence and in the presence of testosterone. While this effect could conceivably contribute to the positive effects of MAA on responses to testosterone (e.g., gene classes IIa, IIc, and IIId; Table 1), it does not explain the inhibitory effects of MAA on responses to testosterone seen in gene classes IIIa and IIIc. Moreover, for many genes in classes IIa, IIc and IIId, where MAA enhances responses to testosterone, the magnitude of the effect of MAA is greater than the observed ~2-fold increase in AR expression. These findings suggest the involvement of other transcription factors in the effects of MAA on testosterone-responsive genes.

FOXO proteins can associate with AR and other nuclear/steroid hormone receptors, leading to either inhibition or enhancement of receptor transcriptional activity [12]. These interactions have the potential to impact the development of hormone-dependent cancers, including prostate, breast and ovarian cancer [12]. Here, we found that FOXO motifs were enriched in 53 genes repressed by testosterone irrespective of whether MAA was present (11% of the genes in TFS group 9.2002), and in 12 genes down-regulated by testosterone, but only when MAA was present, and vice versa (15% of genes in TFS group 12.0022) (Additional file 2, Table S2D). These findings suggest that FOXO factors plays an important role in cellular responses to testosterone and their modulation by MAA. 18 of the potential FOXO targets are involved in transcription regulation, including 6 *Hox *genes (*Hoxb5, Hoxb9, Hoxc6, Hoxc*8, *Hoxd3 *and *Hoxd13) *(Additional file 2, Table S2D). Of note, loss of *Hoxc6 *has been reported to induce apoptosis [50]. Moreover, 16 of the 65 FOXO target genes down regulated by testosterone are associated with developmental processes, as indicated by their GO terms. Based on our microarray signal intensity data, at least three FOXO genes are either highly expressed (*Foxo1*) or moderately expressed in untreated TM3-AR cells (*Foxo6*, *Fox3a)*, suggesting these factors may mediate the effects on FOXO target genes. FOX family genes are primarily regulated through the phosphoinositide-3-kinase (PI3k)-Akt pathway via phosphorylation and nuclear exclusion [11], which is consistent with our earlier finding that the PI3K/Akt pathway is required for the effects of MAA on AR transcriptional activity [22]. Two other transcription factors that are expressed in TM3-AR cells and may be involved in the interactions between testosterone and MAA are LEF/TCF and STAT5. Binding sites for LEF/TCF are significantly enriched in several sets of genes that are regulated by testosterone and MAA, while binding sites for STAT5 are enriched in genes repressed by testosterone (TFS group 9.2002) and in genes whose induction by testosterone was blocked by MAA (TFS group 1.1000) (Table 2; Additional file 3, Table S3B). These findings are consistent with reports that STAT5 and LEF/TCF can modulate AR-regulated gene responses, with STAT5 showing positive interactions with AR [8], and LEF/TCF either repressing or enhancing AR activity [14]. Similarly, our finding that binding sites for MEF2 are enriched in TFS groups responsive to testosterone or MAA (Table 2) is consistent with the finding that binding sequences for MEF2 family transcription factors are commonly found near binding sites for AR, at least in muscle cells [51].

### Possible roles for miRNAs in MAA and testosterone responses

miRNAs are short, ~22 nucleotides long RNAs that generally bind to 3'-UTR sequences of target mRNAs, resulting in post-transcriptional mRNA down regulation and translational repression [52]. Here, we identified several miRNAs whose putative target sites are over-represented in genes responsive to MAA or testosterone, suggesting a possible role for these miRNAs in mediating responses to MAA and and testosterone. Genes in TFS group 1.1000, whose induction by testosterone was blocked by MAA, were enriched in 3'-UTR binding sites for the largest number of miRNAs (Table 2). These include mir-9 and miR-519e, which have been reported to down regulate AR protein [53]. Conceivably, MAA could induce these two miRNAs, which in turn, would down regulate AR protein and functional activity. Two other miRNAs whose binding sites were enriched in the genes of TFS group 1.1000, namely mir-20A and mir-202, are induced in testicular tubules following suppression of FSH and androgen [54], which leads to a block in spermiation. The enrichment of these miRNAs in TFS 1.1000 genes suggests that testosterone may down regulate these miRNAs, which would, in turn, lead to the observed up regulation (de-repression) of the TFS 1.1000 genes with mir-20A and mir-202 sites. Moreover, the inhibition of this gene induction by MAA suggests that MAA may block or perhaps reverse the down regulation of these miRNAs by testosterone. Further study is required to determine the effects of testosterone and MAA on these and other testis-expressed miRNAs, and their impact of spermatogenesis and the toxicities associated with MAA exposure.

### Impact of testosterone and MAA on expression of *CYP *and *GST *genes

CYP (cytochrome P450) and GST (glutathione S-transferase) enzymes metabolize a broad range of endogenous and exogenous compounds. Here, we found that the expression of 20 *CYP *and 12 *GST *genes was affected by either MAA or testosterone (Additional file 2, Table S2E). Nine of these genes were induced by MAA alone (*Cyp2d22, Cyp26a1, Cyp26b1, Gstk1, Gstm6, Gstm7, Gstt2, Mgst2 *and *Mgst3)*, while four genes were induced by MAA but down regulated by testosterone (*Cyp1a1, Cyp2s1, Cyp2f2 *and *Mgst3*). Three CYPs that show female-predominant expression in mouse liver [55] were further induced by testosterone in the presence of MAA compared to testosterone treatment alone (*Cyp2b9, Cyp2b10 and Cyp2b13)*. Further studies are needed to determine whether these enzymes play a metabolic role in MAA modulation of testosterone signaling and/or the detoxification of MAA.

## Conclusions

This study investigated on a genome-wide basis the impact of MAA on AR activity in a cultured Leydig cell model. MAA displayed widespread effects on androgen-responsive genes associated with diverse cellular processes, including apoptosis, ion transport, cell adhesion, phosphorylation and transcription. Binding sites for FOXO, HOX, LEF/TCF, STAT5 and MEF2 family transcription factors, as well as those of several miRNAs, were found to be enriched in various groups of genes regulated by testosterone and/or MAA. These findings highlight the complex interactions, both positive and negative, between androgen- and MAA-responsive genes, and provide insight into molecular mechanisms that may contribute to the toxicities associated with exposure to MAA in testicular cells in vivo.

## Competing interests

The authors declare that they have no competing interests.

## Authors' contributions

GB and DJW conceived and designed the experiments, GB and KS performed the experiments, YZ, GB and DJW analyzed the data and wrote the paper, and DJW managed the overall design and execution of the project. All authors read and approved the final manuscript.

## Supplementary Material

Additional file 1 **Table S1: Mouse primer sequences for all genes analyzed by qPCR**. Click here for file

Additional file 2 **Table S2: Detailed listings, classification and summary of genes that respond to testosterone and/or MAA**. Click here for file

Additional file 3 Table S3: Enrichment analysis of GO terms (A) and motifs (B) Click here for file

Additional file 4 **Figure S1: Networks associated with genes impacted by MAA, as identified by Ingenuity Pathway Analysis**. (A) Network involved in reproductive system disease, small molecule biochemistry and lipid metabolism; (B) Small molecule biochemistry, lipid metabolism and drug metabolism. Dashed arrows indicate regulation of gene expression, arrows with solid lines represent protein-DNA interactions, and solid lines indicate protein-protein interactions. Lines in blue identify processes and factors directly connected to testosterone. Click here for file

Additional file 5 **Table S4: Comparison of gene expression ratios determined by qPCR and microarray analysis for 15 genes representing five TFS groups**. Click here for file

Additional file 6 **Figure S2: Motifs enriched in different TFS groups. Shown are motifs (A) and miRNAs (B) with enrichment p-value < 0.001 in at least one TFS group and TFS groups which have at least one motif enriched with *p *< 0.001 are selected**. TFS numbers are shown on the top of the heat map. Please refer to Additional file 2, Table S2C for the biological description of each TFS group. Numbers on the top of the color bar represent -log10 *P *value, with higher numbers indicating greater enrichment. Click here for file
