# Supplementary figures and images for: Complex modulation of androgen responsive gene expression by methoxyacetic acid
Source: Reprod Biol Endocrinol. 2011 Mar 31;9:42. doi: 10.1186/1477-7827-9-42 (PMC3083340; doi:10.1186/1477-7827-9-42)

Figure S1A

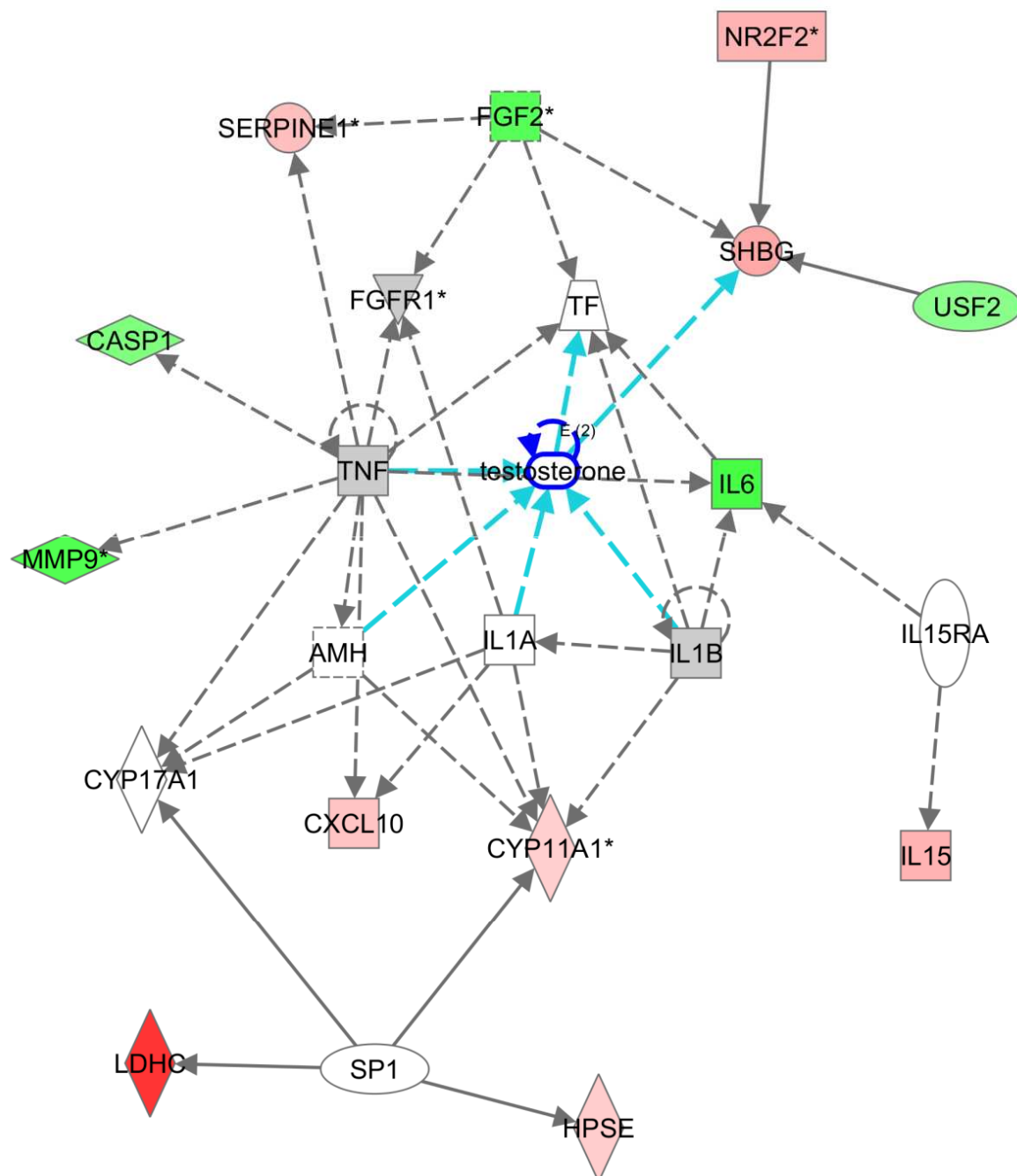

Figure S1B

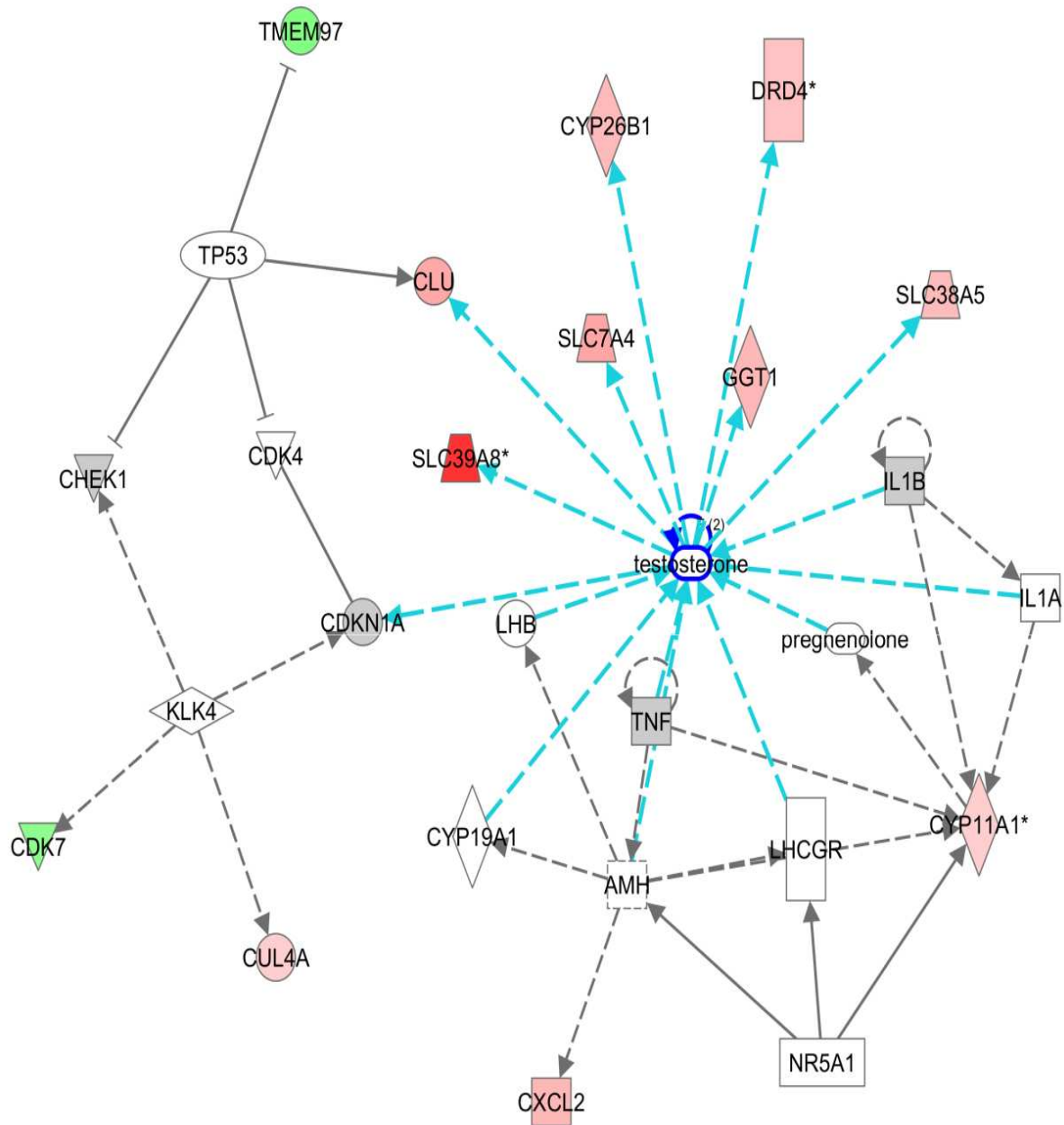

Supplement: Additional file 4 — Figure S1: Networks associated with genes impacted by MAA, as identified by Ingenuity Pathway Analysis. (A) Network involved in reproductive system disease, small molecule biochemistry and lipid metabolism; (B) Small molecule biochemistry, lipid metabolism and drug metabolism. Dashed arrows indicate regulation of gene expression, arrows with solid lines represent protein-DNA interactions, and solid lines indicate protein-protein interactions. Lines in blue identify processes and factors directly connected to testosterone. [file 1477-7827-9-42-S4.PDF]

Figure S2A

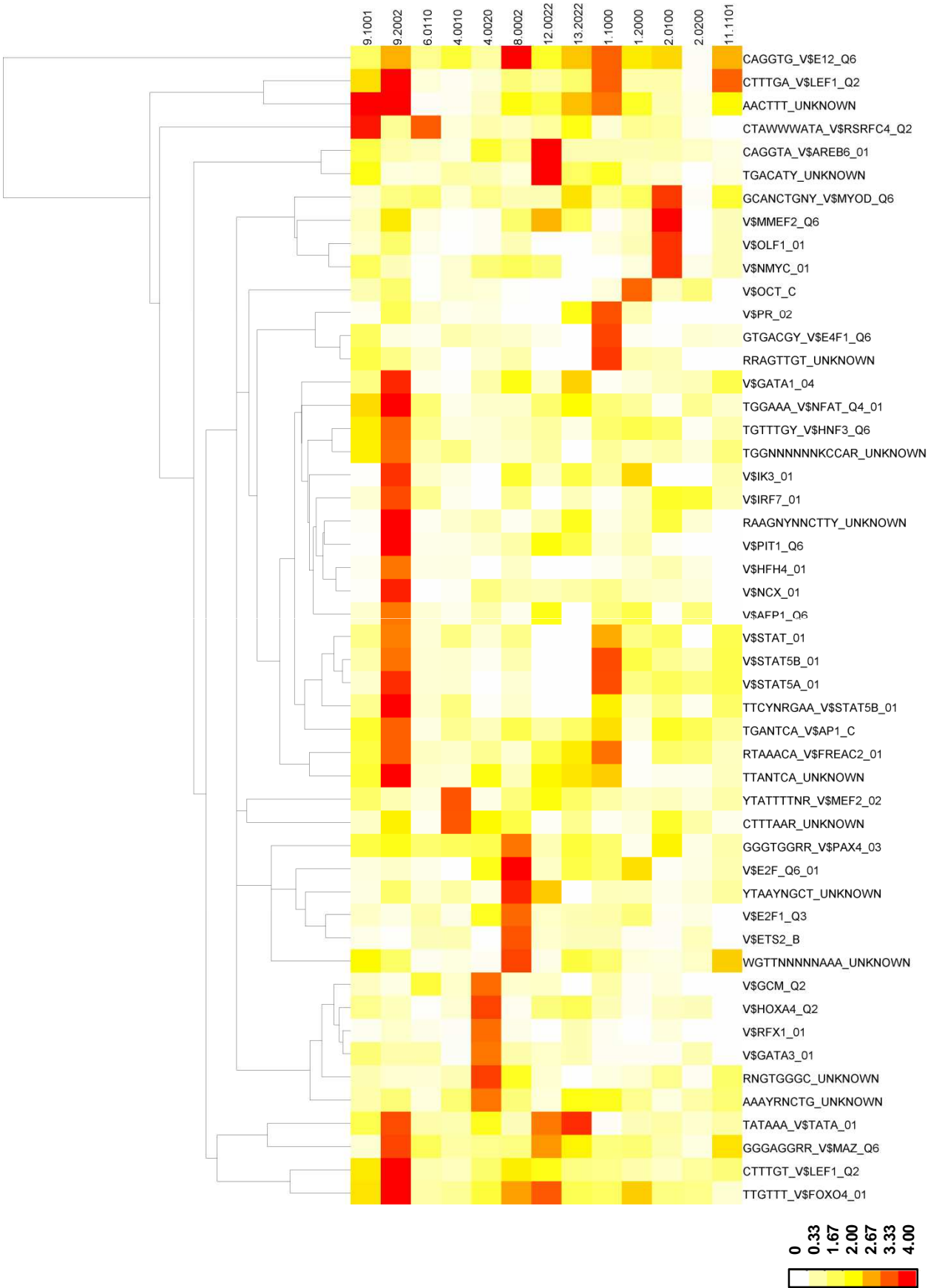

Figure S2B

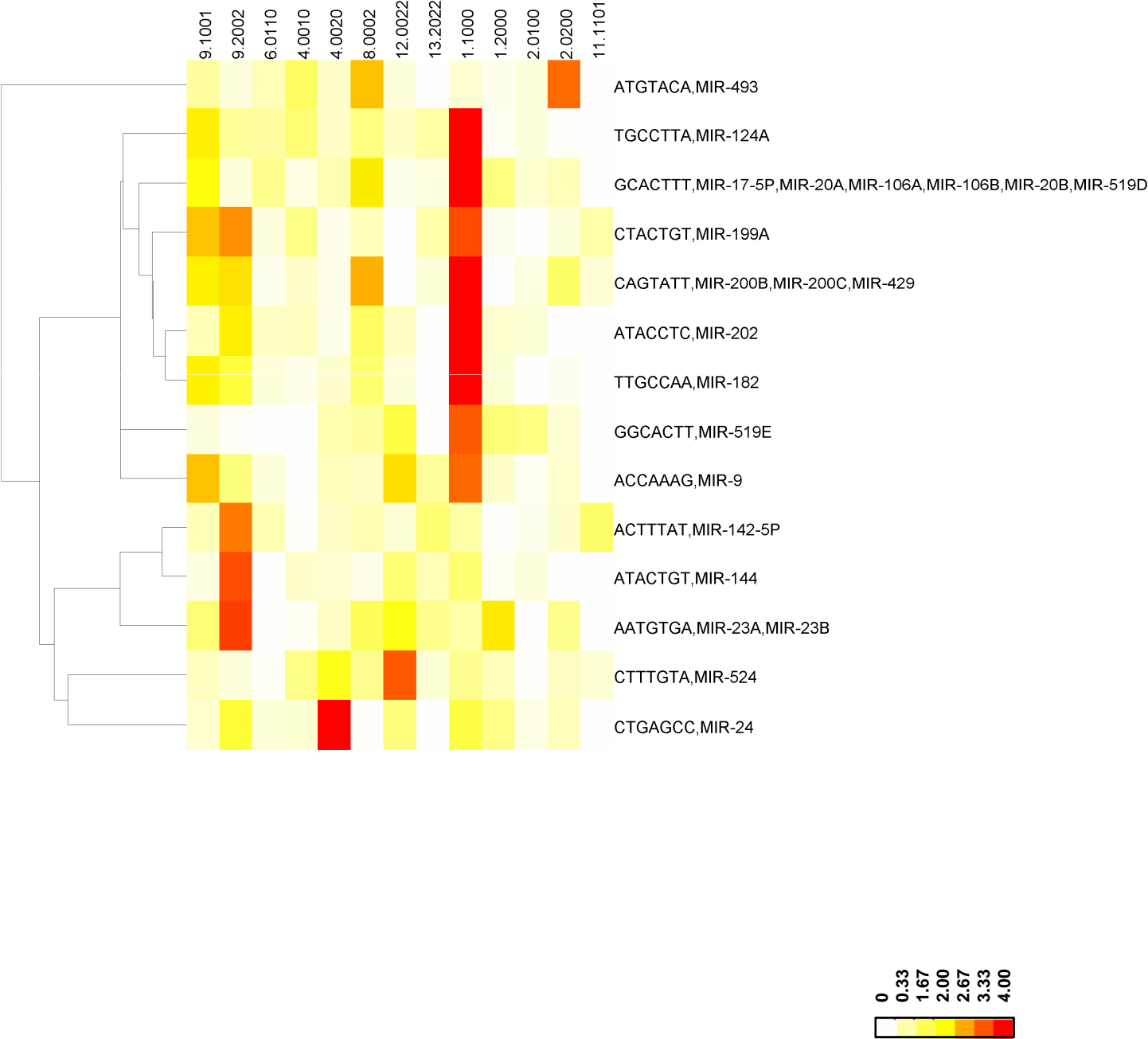

Supplement: Additional file 6 — Figure S2: Motifs enriched in different TFS groups. Shown are motifs (A) and miRNAs (B) with enrichment p-value < 0.001 in at least one TFS group and TFS groups which have at least one motif enriched with p < 0.001 are selected. TFS numbers are shown on the top of the heat map. Please refer to Additional file 2, Table S2C for the biological description of each TFS group. Numbers on the top of the color bar represent -log10 P value, with higher numbers indicating greater enrichment. [file 1477-7827-9-42-S6.PDF]
